# Ciprofloxacin Electrochemical Sensor Using Copper–Iron Mixed Metal Oxides Nanoparticles/Reduced Graphene Oxide Composite

**DOI:** 10.1021/acsomega.3c06705

**Published:** 2024-05-21

**Authors:** Jedsada Chuiprasert, Sira Srinives, Narin Boontanon, Chongrak Polprasert, Nudjarin Ramungul, Apisit Karawek, Suwanna Kitpati Boontanon

**Affiliations:** † Graduate Program in Environmental and Water Resources Engineering, Department of Civil and Environmental Engineering, Faculty of Engineering, Mahidol University, Salaya, Phuttamonthon, Nakhon Pathom 73170, Thailand; ‡ Nanocomposite Engineering Laboratory (NanoCEN), Department of Chemical Engineering, Faculty of Engineering, Mahidol University, Salaya, Phuttamonthon, Nakhon Pathom 73170, Thailand; § Faculty of Environment and Resource Studies, Mahidol University, Salaya, Phuttamonthon, Nakhon Pathom 73170, Thailand; ∥ Department of Civil Engineering, Faculty of Engineering, Thammasat University, Khlong Nueng, Khlong Luang, Pathum Thani 12120, Thailand; ⊥ National Metal and Materials Technology Center, National Science and Technology Development Agency, Khlong Nueng, Khlong Luang, Pathum Thani 12120, Thailand; # Graduate School of Global Environmental Studies, Kyoto University, Yoshida-Honmachi, Sakyo-ku, Kyoto 606-8501, Japan

## Abstract

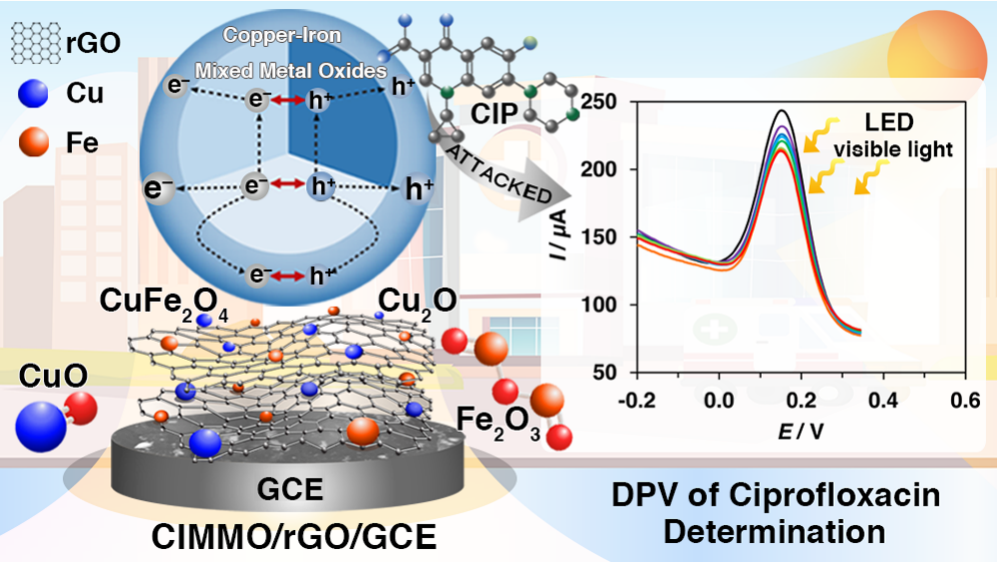

The harmful effects of antibiotic proliferation on the environment and its persistent nature are urgent global problems. Ciprofloxacin (CIP) is a fluoroquinolone-class antibiotic agent used widely to treat pathogen-related diseases in humans and animals. Its excretion into surface water causes antibiotic resistance in microbes, resulting in difficult-to-treat or untreatable infectious diseases. This study developed a simple and efficient electrochemical sensor to detect CIP. Hydrothermal chemistry was utilized to synthesize an electrophotocatalytic composite of copper–iron mixed metal oxides (CIMMO) on reduced graphene oxide (rGO) (CIMMO/rGO). The composite was employed in an electrochemical sensor and exhibited outstanding performance in detecting CIP. The sensor was operated in differential pulse voltammetry (DPV) mode under light source illumination. The sensor yielded a linear response in the concentration range of 0.75 × 10^–9^–1.0 × 10^–7^ mol L^–1^ CIP and showed a limit of detection (LOD) of 4.74 × 10^–10^ mol L^–1^. The excellent sensing performance of the composite is attributable to the synergic effects between CIMMO nanoparticles and rGO, which facilitate photoinduced electron–hole separation and assist in the indirect electrochemical reactions/interactions with CIP.

## 1 Introduction

Antibiotic resistance (ABR) is a global issue related to the overuse and misuse of antibiotics, drugs that treat bacterial infections. Such inappropriate use of antibiotics results in their contamination in surface water and groundwater and the potential development of superpathogens with ABR.^1^ Ciprofloxacin (CIP, C_17_H_18_FN_3_O_3_), a synthetic, second-generation, fluoroquinolone-class antibiotic, has been used to treat Gram-negative and Gram-positive infections in humans and animals.^2^ Overuses of CIP have led to pollution and contamination of excessive and unmetabolized CIP in water sources, contributing to the ABR issue. CIP was detected at ng L^–1^ to mg L^–1^ levels in surface, ground, and drinking water as reported in recent studies.^3,4^ Conventional techniques, including capillary electrophoresis, gas chromatography (GC), and high-performance liquid chromatography with tandem mass spectrometry (HPLC−MS/MS) are currently used as standard methods for the detection of CIP.^5^ Although the detection results from these conventional techniques are precise and reliable, they rely heavily on costly equipment and complex procedures. Thus, there is a need for an easy-to-use, mobile, sensitive, and selective device for CIP detection in water.

Electrochemical sensors are a group of low-cost, lightweight, and mobile devices that can serve as an ideal platform for the detection of antibiotics.^6−9^ The devices utilize electrochemical reactions and interactions between a target chemical and a sensitive element to generate the sensing signal.^10^ Bagheri et al. synthesized an electrochemical sensor using magnetic multiwalled carbon nanotubes (MMWCNTs) and a molecularly imprinted polymer (MIP) as a sensitive element for CIP detection. They reported a limit of detection (LOD) of 1.7 × 10^–9^ mol L^–1^.^11^ Li et al. used an MIP of gold nanoparticle/black phosphorus nanocomposites for selective detection of pefloxacin. The composites demonstrated a broad linear detection range with an LOD of 0.80 × 10^–9^ mol L^–1^.^12^ Our group reported the production of an electrochemical sensor utilizing an MIP composite of polyaniline, poly(*o*-phenylenediamine), and reduced graphene oxide (rGO) as the sensitive element. The sensor realized an LOD of 5.28 × 10^–11^ mol L^–1^ for CIP detection with adequate reusability.^13^

Metals and metal oxides are outstanding candidates for sensitive elements because of their stability, ease of processing, and well-explored characteristics.^14^ Many metals, metal oxides, and mixed metal oxide composites, such as bismuth oxychloride,^15^ cobalt ferrite,^16^ copper–titanium dioxide (TiO_2_),^17^ and TiO_2_–graphene,^18^ have been demonstrated for their electroactivity. Copper–iron mixed metal oxides (CIMMO) are combinations of iron and copper oxides with enhanced photoactivity and redox capabilities.^19^ Gholivand et al. synthesized a copper–iron oxides/TiO_2_ composite and used it as part of an electrochemical sensor for metformin detection in pharmaceutical products and human urine.^20^ Iron oxides/TiO_2_ was described as the main sensitive element for the sensor, whereas the copper oxides served as a dopant that promoted metformin adsorption and charge transfer. The working group of Khalilzadeh modified a carbon screen-printed electrode with an iron (II, III) oxides (Fe_3_O_4_)/cellulose nanocrystals/copper nanocomposite.^21^ The electrode was sensitive to venlafaxine while operated in differential pulse voltammetry (DPV) mode.

Graphene oxide (GO), a two-dimensional (2D) carbon nanostructure, is a layered carbon sheet with structural defects from functional groups such as carboxyl, hydroxyl, and epoxy. GO can be reduced based on chemical or thermal reduction to rGO, in which a portion of the functional groups is removed. rGO provides the electrical properties of a semiconductor and is a good sensitive element or charge-transfer promotor in a sensor device.^22,23^ GO can be composited with metals and metal oxides and becomes a mixed metal oxides/rGO composite. Side reactions involve the conversion of GO to rGO due to reduction reactions occurring during chemical and thermal treatment. The mixed metal oxides can be deposited as nanoparticles on the structural defects of rGO, immobilized, and separated from one another with high surface reactivity for reactions.^24−26^ In addition, mixed metal oxides/rGO interfaces assist in stabilizing active radicals and intermediates and promoting electron–hole separation, which enhances the catalytic activities of composites.

In this research, we synthesized the CIMMO/rGO composite following a one-step hydrothermal technique for use as a sensitive element in an electrochemical sensor. The sensor was operated in the DPV mode to detect CIP in water solutions. The solutions were prepared by using ultrapure water (UPW) or surface water. The reusability, reproducibility, and stability of the sensor were also examined to assess its practical usability.

## 2 Results and Discussion

### 2.1 Physical and Chemical Characterizations

#### 2.1.1 Micro X-ray Fluorescence (μXRF) Analysis

**Figure 1:**
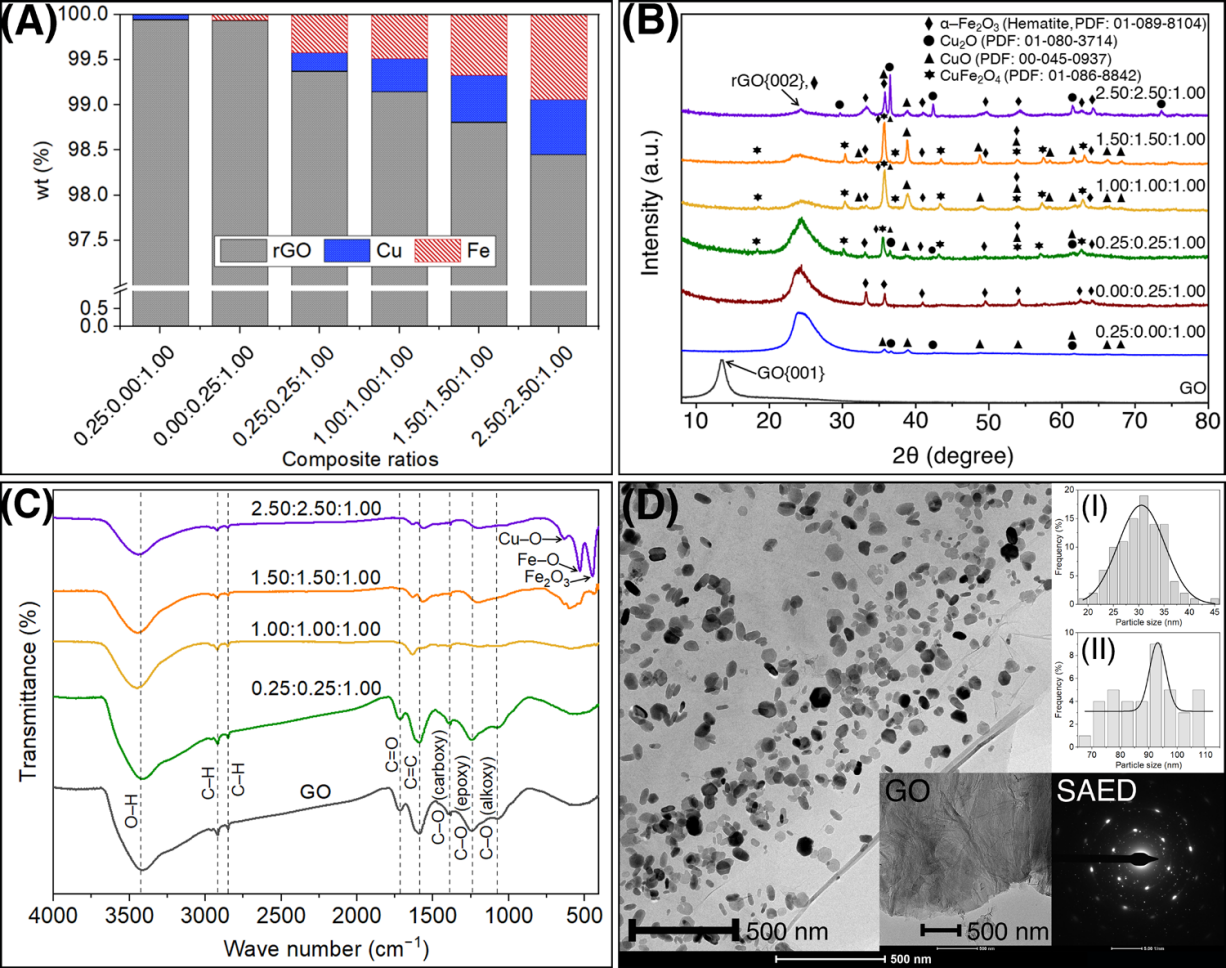
Analytical results from the composite samples: XRF analysis (A); XRD patterns (B); FT**-**IR spectra (C); TEM of 0.25:0.25:1.00 CIMMO/rGO (D), GO (D, Inset), average size distributions of CIMMO nanoparticles with a Gaussian-fitting curve (D, I inset) and (D, II inset), and SAED pattern (D, Inset).

The mass compositions of the composite samples were analyzed by using μXRF. The carbon element (C) from rGO was interpreted from residual data using the fundamental parameter method (Figure 1A). The 0.25:0.00:1.00 CIMMO/rGO composite sample was obtained from 2.5 mg of copper nitrate trihydrate (Cu(NO_3_)_2_·3H_2_O) and 1.0 mg of GO with no ferric chloride (FeCl_3_). The composite displayed 0.06%w/w copper element (Cu) incorporated with rGO (99.04%w/w), with no iron element (Fe). The 0.00:0.25:1.00 CIMMO/rGO sample was synthesized using 2.5 mg of FeCl_3_ with 1.0 mg of GO (0.00:0.25:1.00 Cu:Fe:GO). The composite exhibited 0.06%w/w Fe on 99.04%w/w rGO. Analysis of the 0.25:0.25:1.00 CIMMO/rGO revealed 0.21, 0.42, and 99.37%w/w for Cu, Fe, and rGO, respectively. Increasing the proportions of the metal precursors to 1.00:1.00:1.00 and 1.50:1.50:1.00 (1.00:1.00:1.00 and 1.50:1.50:1.00 CIMMO/rGO) produced composites with 0.37%w/w Cu, 0.48%w/w Fe, and 99.15%w/w rGO and 0.52%w/w Cu, 0.67%w/w Fe, and 98.81%w/w rGO, respectively. Analysis of the 2.50:2.50:1.00 CIMMO/rGO provided mass compositions of 0.61, 0.94, and 98.45%w/w for Cu, Fe, and rGO, respectively.

#### 2.1.2 X-ray Diffraction (XRD) Analysis

The crystal structures of the composites prepared from different ratios of CIMMO precursors were analyzed by XRD (Figure 1B). GO showed a single diffraction peak at 13.5°, which can be ascribed to the {001} plane of GO structures.^27^ All composite samples revealed a peak at 24.4°, which correlated to the {002} plane of the rGO. The result confirmed the side reaction that converted GO to rGO during hydrothermal. The 0.25:0.00:1.00 CIMMO/rGO composite presented crystalline phases of cupric oxide (CuO) and cuprous oxide (Cu_2_O). The peak positions at 35.7°, 38.9°, 48.9°, 53.9°, 61.6°, 66.3°, and 68.1° corresponded to the {001}, {111}, {−202}, {020}, {−113}, {−311}, and {−220} crystal planes of CuO (PDF: 00-045-0937). The other three characteristic peaks at 36.7°, 42.6°, and 61.6° were attributed to the {111}, {200}, and {220} crystal planes of Cu_2_O (PDF: 01-080-3714), respectively. The diffraction pattern for 0.00:0.25:1.00 CIMMO/rGO showed peaks at 33.2°, 35.7°, 40.9°, 49.6°, 54.2°, 62.4°, and 64.2°, which correlated to the {104}, {110}, {113}, {024}, {116}, {214}, and {300} crystal planes of the hematite iron oxide (α-Fe_2_O_3_) phases (octahedral and rhombohedral) (PDF: 01-089-8104), respectively. The 0.25:0.25:1.00, 1.00:1.00:1.00, and 1.50:1.50:1.00 CIMMO/rGO compositions provided similar diffraction patterns, showing signals for three crystalline phases: copper ferrite (CuFe_2_O_4_), α-Fe_2_O_3_, and CuO. Additional peaks from 0.25:0.25:1.00 and 2.50:2.50:1.00 CIMMO/rGO were observed at 36.7°, 42.6°, and 61.6°, which represented the {111}, {200}, and {220} planes of Cu_2_O, respectively. The peaks at 18.2°, 30.3°, 35.8°, 38.7°, 43.3°, 54.1°, 57.3°, and 63.2° corresponded to the {111}, {220}, {311}, {222}, {400}, {422}, {511}, and {440} planes of CuFe_2_O_4_ (PDF: 01-086-8842).

#### 2.1.3 Fourier Transform Infrared Spectroscopy (FT-IR) Analysis

FT**-**IR was used to analyze the chemical functionality (Figure 1C). The GO spectra peaked at 3421 cm^–1^, indicating the presence of O–H stretching group and adsorbed water molecules. The peaks at 2915 and 2850 cm^–1^ correspond to alkyl C–H stretching. The IR transmittance peaks at 1720, 1590, 1390, 1240, and 1080 cm^–1^ correlated to the −C=O stretching of carbonyl, C=C stretching, C–O stretching of carboxyl, C–O stretching of epoxy, and C–O stretching of alkoxy.^28,29^ Some GO IR transmittance peaks, revealing O–H stretching, C–H stretching, C=C stretching, and C–O stretching, were observed on the composites spectra. The O–H stretching signal at 3421 shifted to 3428 cm^–1^, suggesting interactions between copper and iron mixed metal oxides and −OH groups.^30^ The IR spectrum of 0.25:0.25:1.00 CIMMO/rGO appeared to be identical to the GO spectrum but with reduced peak intensity. The results demonstrated good CIMMO adhesion onto rGO and partial removal of the chemical functionalities. For 1.00:1.00:1.00, 1.50:1.50:1.00, and 2.50:2.50:1.00 CIMMO/rGO, IR transmittance peak intensities were further reduced. An additional peak appeared at 633 cm^–1^ and was interpreted as Cu–O from CuO/Cu_2_O species.^31^ The 2.50:2.50:1.00 CIMMO/rGO showed two additional characteristic peaks at 533 and 447 cm^–1^, which corresponded to Fe–O stretching from the Fe_2_O_3_.^32^ As the amount of rGO in the CIMMO/rGO composite increased, the intensity of the Cu–O and Fe–O stretching bands decreased. Therefore, it was clear that the CIMMO nanoparticles were attached and altered the IR transmittance peaks of rGO.

#### 2.1.4 Morphology and Size Distribution Analysis

Transmission electron microscopy (TEM) images revealed the morphology of the CIMMO nanoparticles on the rGO sheet (Figure 1D). GO (Figure 1D, Inset) displayed a sheet-like structure with wrinkles and micrometer-scale dimensions. In 0.25:0.25:1.00 CIMMO/rGO, the CIMMO nanoparticles were dispersed homogeneously on the rGO sheet, showing two average size distribution histograms with a Gaussian-fitting curve of 30.51 ± 9.22 nm (Figure 1D, I inset) and 93.12 ± 5.73 nm (Figure 1D, II inset), respectively. The selected area electron diffraction (SAED) provided clear ring patterns (Figure 1D, inset), indicating polycrystal morphologies with large grain sizes for the nanoparticles. We located four types of nanostructures in the TEM images: CuO/Cu_2_O nanorods, α-Fe_2_O_3_ octadecahedra, α-Fe_2_O_3_ rhombohedra, and CuFe_2_O_4_ irregular nanoparticles. The CuO/Cu_2_O nanorods had a width and length of 30.51 ± 9.22 and 62.73 ± 8.11 nm, respectively (Figure S1A,B). The α-Fe_2_O_3_ appeared as crystalline nanoparticles with octadecahedral structures^33^ and a size of 85.46 ± 28.34 nm (Figure S1C). The α-Fe_2_O_3_ rhombohedral^34^ nanostructure exhibited a width of 86.07 ± 28.39 nm (Figure S1D) and a length of 93.12 ± 5.73 nm (Figure S1E). The CuFe_2_O_4_ nanoparticles had an irregular shape^35^ with an average width of 52.10 ± 20.03 nm (Figure S1F) and a length of 86.92 ± 18.58 nm (Figure S1G).

#### 2.1.5 X-ray Photoelectron Spectroscopy (XPS) Analysis

**Figure 2:**
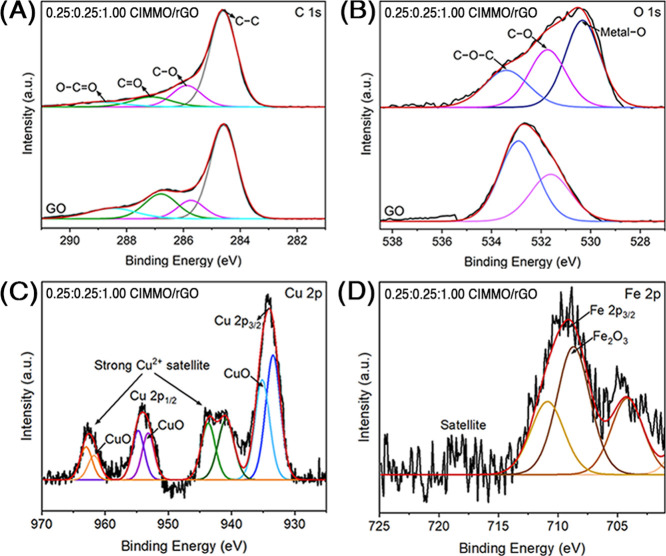
XPS narrow scan of GO and 0.25:0.25:1.00 CIMMO/rGO composite, focusing on C 1s (A), O 1s (B), Cu 2p (C), and Fe 2p (D).

XPS was utilized for the chemical composition analysis of GO and 0.25:0.25:1.00 CIMMO/rGO composite. For GO, the XPS survey scan (Figure S2) showed significant peaks at 295, 550, and 991 eV, representing carbon (C 1s) and oxygen (O 1s and O KLL) species. The C 1s narrow scan (Figure 2A) presented binding peaks at 284.6, 285.8, 287.1, and 288.7 eV, which correlated to C–C, C–O, C=O, and O–C=O species.^36^ The O 1s narrow scan (Figure 2B) displayed peaks at 531.8 and 533.3 eV, which were associated with C–O and C–O–C bonds in the GO structure. For the CIMMO/rGO composite, the wide scan exhibited similar binding peaks as observed from the GO. However, an additional peak was noticed from the O 1s narrow scan at 530.3 eV, representing the Metal–O binding species.^37,38^ The peak intensities at 533.7 and 531.6 eV, which indicated the presence of C–O–C and C–O species, were significantly reduced compared to GO.^36^ The reduction in signal intensity from the functionalities could be attributed to a partial reduction reaction during the hydrothermal treatment for the composite’s production. Figure 2C shows a narrow scan for Cu 2p of the composite, indicating Cu(0) and CuO/Cu_2_O forms as major copper species. The Cu 2p_3/2_ narrow scan provides peaks at 933.4 and 935.1 eV, which were ascribed to Cu_2_O/Cu^39^ and CuO species.^40^ The Cu 2p_1/2_ scan revealed binding energy peaks at 953.1 and 954.7 eV, corresponding to the CuO species. Two binding energy peaks for the Cu^2+^ satellites were identified at 942.2 and 962.5 eV, indicating the formation of copper ferrite alloys, such as Cu_2_O–CuFe_2_O_4_.^41^ The interaction between Cu^2+^ and CIP is promoted by complexation with metal ions.^42^ The Fe 2p narrow scan (Figure 2D) displayed a firm peak that corresponded to Fe 2p_3/2_ at 710.9 eV. The peak represented the Fe^2+^/Fe^3+^ species in the composite.^33^ It has been reported in the literature that the combination of Cu^+^/Cu^2+^ and Fe^2+^/Fe^3+^ assists in the redox reactions and could be a factor for the synergic effects between CIMMO/rGO and CIP.^43^

#### 2.1.6 Brunauer–Emmett–Teller (BET) Surface Area and Pore Volume Analysis

**Figure 3:**
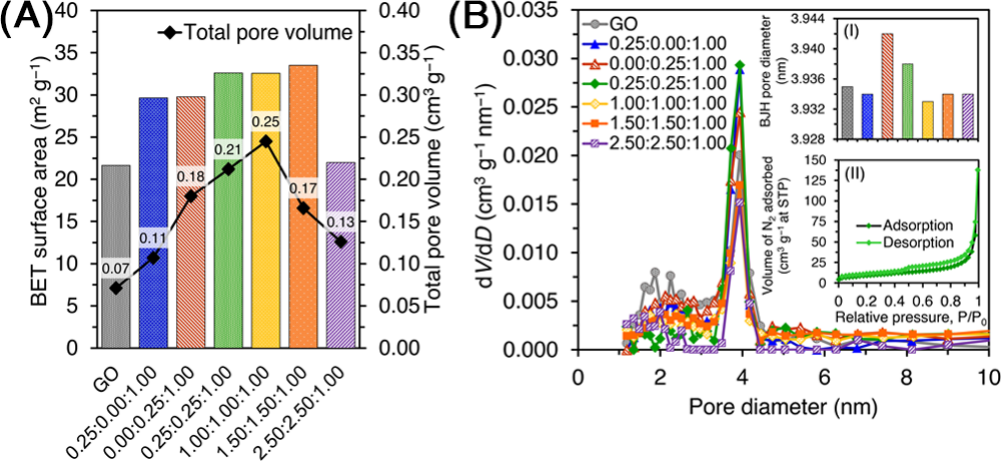
BET surface area and BJH pore diameter (A); pore volume distributions of composites (B) and N_2_ adsorption–desorption isotherms of the 0.25:0.25:1.00 CIMMO/rGO (B, Inset).

The Barrett–Joyner–Halenda (BJH) pore diameter and the pore volume of the GO and composites are shown in Figure 3A. GO had a surface area of 21.63 m^2^ g^–1^, a pore diameter of 3.94 nm, and a pore volume of 0.07 cm^3^ g^–1^ (Figure 3B). The 0.25:0.00:1.00 CIMMO/rGO provided a 37% increment in BET surface (29.64 m^2^ g^–1^) but an equivalent pore size (3.93 nm) and pore volume (0.11 cm^3^ g^–1^) compared to GO. The 0.00:0.25:1.00 CIMMO/rGO showed an increased BET surface (29.77 m^2^ g^–1^) with an equivalent pore size (3.94 nm) and pore volume (0.18 cm^3^ g^–1^). The 0.25:0.25:1.00, 1.00:1.00:1.00, and 1.50:1.50:1.00 CIMMO/rGO composites yielded surface areas of 32.61, 32.58, and 33.52 m^2^ g^–1^, pore sizes of 3.94, 3.94, and 3.93 nm, and pore volumes of 0.21, 0.25, and 0.17 cm^3^ g^–1^, respectively. The 2.50:2.50:1.00 CIMMO/rGO presented a surface area of 22.50 m^2^ g^–1^, a pore size of 3.93 nm, and a pore volume of 0.13 cm^3^ g^–1^. The BET results agreed with the XRD results as the increased BET surface area and pore volume were attributable to the incorporation of CIMMO on GO. The pore sizes remained relatively constant with regard to the metal/metal oxide decoration, suggesting a transformation of the larger pores of the composites to deeper and narrower pores. This phenomenon was discontinued for 1.50:1.50:1.00 and 2.50:2.50:1.00 CIMMO/rGO as surface areas and pore volumes both decreased. The decrease could be attributed to the overdeposition of metal/metal oxide on the rGO structure. The N_2_ adsorption–desorption isotherms (Figure 3B, Inset) obtained from the 0.25:0.25:1.00 CIMMO/rGO indicated a type-IV isotherm with a broad H4 hysteresis loop according to the recommendation of the International Union of Pure and Applied Chemistry (IUPAC). The isotherm demonstrated the initial formation of monolayer–multilayer adsorption in the mesopores, followed by hysteresis capillary condensation inside the pores. The H4 hysteresis loop was associated with an adsorbent with slit-shaped mesopores such as interfaces between CIMMO nanoparticles and CIMMO and rGO in the composite.

### 2.2 Electrochemical Characteristics of CIMMO/rGO/GCE

#### 2.2.1 Determination of the Electroactive Surface Area

**Figure 4:**
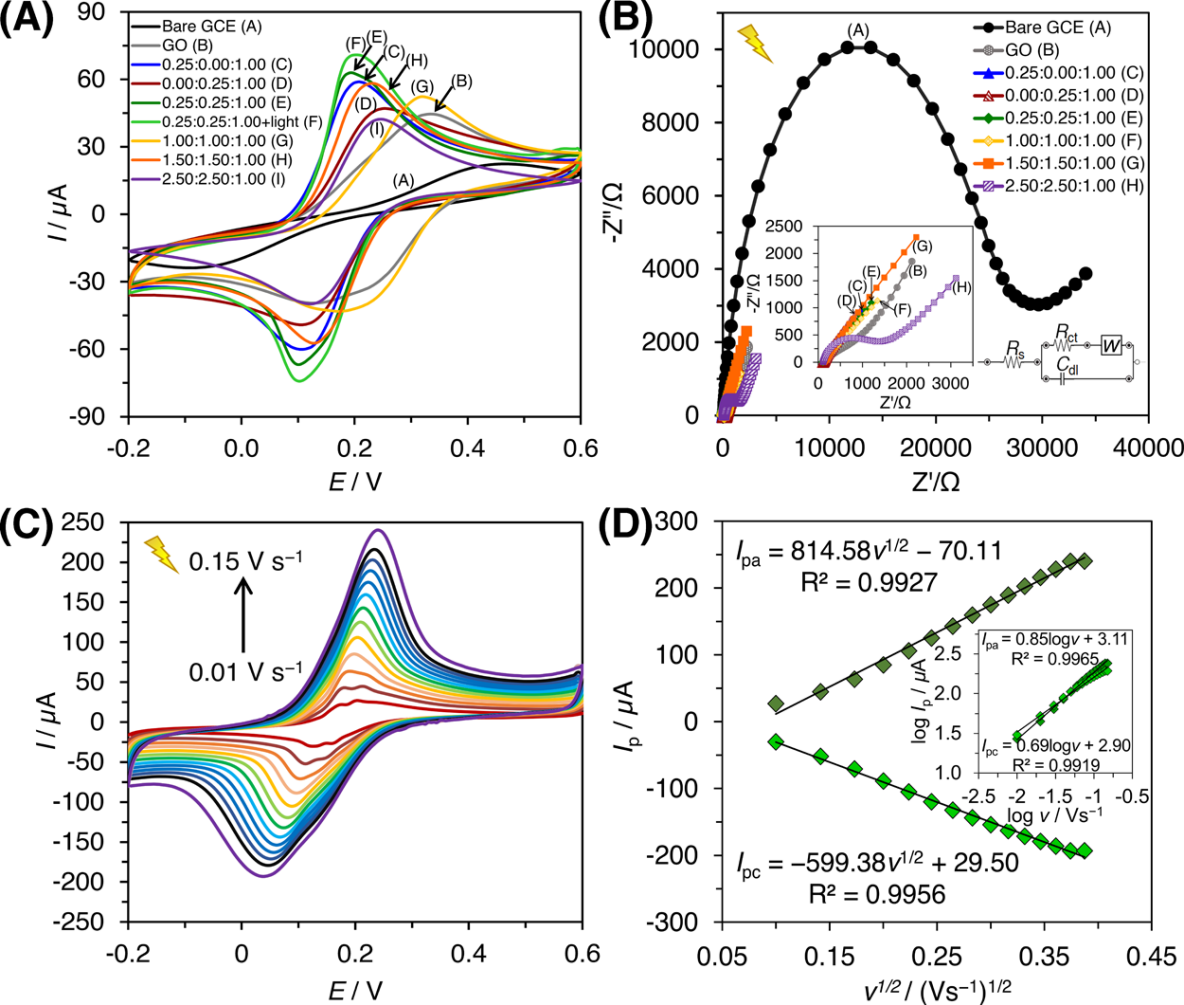
CV of the modified electrodes in [Fe(CN)_6_]^3–/4–^ redox mediator (A); EIS Nyquist plots of the modified electrodes (0.1–100,000.0 Hz) (B) and the Randles equivalent circuit (B, Inset); CV plot of 0.25:0.25:1.00 CIMMO/rGO/GCE (0.01–0.15 V s^–1^ scan rate) (C); *I*_p_ vs ν^1/2^ plot (D) and Log *I*_p_ vs log *v* plot (D, Inset) (*n* = 3).

The electrochemical characteristics of the modified electrodes were investigated using the cyclic voltammetry (CV) technique in a solution containing ferri/ferrocyanide redox mediator ([Fe(CN)_6_]^3–/4–^) (Figure 4A). Experiments were conducted in a closed chamber, where the three electrodes were employed in a quartz cell and operated with or without light illumination. The effective surface area of a modified electrode was determined using the Randles–Sevcǐk equation (eq 1)
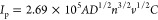
1where *I*_p_ is the peak current, *A* is the electroactive area (cm^2^), *D* is the diffusion coefficient (4.0 × 10^–6^ cm^2^ s^–1^), *n* is the number of electrons, *v* is the scan rate (V s^–1^), and *C* is the concentration of [Fe(CN)_6_]^3–/4–^ (mol cm^–3^). Without light illumination, the area was 0.04 cm^2^ for a bare glassy carbon electrode (GCE) but increased to 0.08 cm^2^ for a GO-modified GCE. The 0.25:0.25:1.00 CIMMO/rGO/GCE displayed an area of 0.11 and 0.12 cm^2^ in the absence and presence of light illumination, respectively. The electroactive areas for 0.25:0.00:1.00, 0.00:0.25:1.00, 1.00:1.00:1.00, 1.50:1.50:1.00, and 2.50:2.50:1.00 CIMMO/rGO/GCE were 0.10, 0.08, 0.10, 0.10, and 0.07 cm^2^, respectively. The areas per gram of catalyst (g_CAT_) for GO/GCE and 0.25:0.00:1.00, 0.00:0.25:1.00, 0.25:0.25:1.00, 1.00:1.00:1.00, 1.50:1.50:1.00, and 2.50:2.50:1.00 CIMMO/rGO/GCE were 5.90, 6.90, 5.87, 7.70, 6.97, 6.95, and 4.98 m^2^ g_CAT_^–1^, respectively. The electroactive area of the composite was 2.5 times less than that of the BET surface area, which could be attributed to parts of the composite pores not being electroactive or reachable by the electrolyte.

#### 2.2.2 Electrochemical Impedance Spectroscopy (EIS)

EIS was used to study the electrochemical characteristics of the composite electrode in the [Fe(CN)_6_]^3–/4–^ redox mediator. The data were fitted by using an equivalent circuit function on the electrochemistry workstation. The notation *C*_dl_ denotes the double-layer capacitance, *R*_s_ denotes the electrolyte resistance, *W* denotes the Warburg impedance, and *R*_ct_ denotes the electron transfer resistance obtained from the semicircular diameter of the impedance. The simulated circuit connected *R*_s_ in parallel with *C*_dl_, *R*_ct_, and *W*. *R*_s_ was relatively constant for all of the tested samples as the same electrolyte composition was used. The Nyquist plots (Figure 4B) showed an *R*_ct_ of 26,334.39 Ω for the bare GCE but only 63.69 Ω for the GO/GCE. The *R*_ct_ of the 0.00:0.25:1.00 CIMMO/rGO composite was even lower, equaling 4.73 Ω due to improved charge transfer ability and electroactivity from CIMMO and rGO.^24^ The *R*_ct_ decreased to 1.33, 2.60, and 3.23 Ω for the 0.25:0.25:1.00, 1.00:1.00:1.00, and 1.50:1.50:1.00 CIMMO/rGO composites, respectively. The 2.50:2.50:1.00 CIMMO/rGO exhibited an *R*_ct_ of 1,198.81 Ω, which was in agreement with the lessened electroactive surface areas noticed in the previous test. The results indicated that 0.25:0.25:1.00 CIMMO/rGO gave the lowest *R*_ct_ with the highest electroconductivity.

#### 2.2.3 Electrochemical Characteristics of the Modified Electrode

The electroactivity of the composite was studied by using a cyclic voltammogram in the [Fe(CN)_6_]^3–/4–^ medium (Figure 4C). The 0.25:0.25:1.00 CIMMO/rGO/GCE was employed in the electrochemical cell and illuminated. The applied potential was scanned within a −0.2 to 0.6 V window with a scan rate varying from 0.01 to 0.15 V s^–1^. The CV curve provided redox potential peaks at 0.03 (reduction) and 0.20 V (oxidation), which tended to shift for a higher scan rate. The shift in the peaks suggested irreversible redox reactions at the working electrode. A plot of anodic peak current (*I*_pa_) versus scan rate (*v*^1/2^) (Figure 4D) revealed the correlation of *I*_pa_ (μA) = 814.58 *v*^1/2^ (V s^–1^)^1/2^ – 70.11, with *R*^2^ = 0.9927. The cathodic current peak (*I*_pc_) correlation was *I*_pc_ (μA) = −599.38 *v*^1/2^ (V s^–1^)^1/2^ + 29.50, with *R*^2^ = 0.9956. The Log *I*_p_ versus log *v* plots revealed a slope of 0.85 and 0.69 for *I*_pc_–*v* and *I*_pa_–*v*, respectively. The slopes fell within 0.5–1.0 windows, addressing the combined adsorption and diffusion-controlled mass transport.^44^ The peak potential (*E*_P_) follows the correlation from eq 2(45):
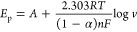
2where *A* is a constant related to the formal electrode potential (*E*_0_), α is the transfer coefficient (α = 0.5) representing the effect of electrochemical potential, *n* is the number of electrons involved in the rate-controlling step, and *R, T*, and *F* are the gas constant, temperature, and Faraday constant, respectively. The *n* values were determined to be 0.6 and −0.8 for anodic and cathodic reactions, suggesting one proton and one electron transfer behavior at the electrode, which was consistent with the results of previous studies.^46^ The composite displayed a magnetic property as it was attracted easily by an external magnetic field (Figure S3A), which suggested a strong electroactivity of the material.^47^

### 2.3 CIP Detection Experiments

#### 2.3.1 Effect of Solution pH on Sensing Responses

For the CIP detection experiments, we used phosphate buffer solution (PBS) containing 1.0 × 10^–6^ mol L^–1^ CIP and evaluated the influence of solution pH within a pH window of 4–8 (Figure S3B). The tests were conducted in DPV mode, with potential scanning from +0.4 to +1.4 V. As the solution pH increased from 4 to 6, the electrochemical responses increased from 150 to 165 μA. The signal stabilized for a CIP solution pH of 6.5 but dropped significantly at 8.0 due to proton scarcity in the alkaline solution.^11^ The correlation was linear in the pH 4–8 range (eq 3), showing a regression (*R*^2^) of 0.95.
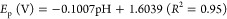
3

The slope was determined to be −0.1007 V pH^–1^, deviating from the theoretical Nernstian value of −0.059 V pH^–1^. The deviation could be attributable to charge losses to other active radicals and differences in experimental conditions, such as electrolyte concentration and temperature.^48^ Based on the findings, a pH 6.5 solution was selected for subsequent investigations.

#### 2.3.2 CIP Detection

**Figure 5:**
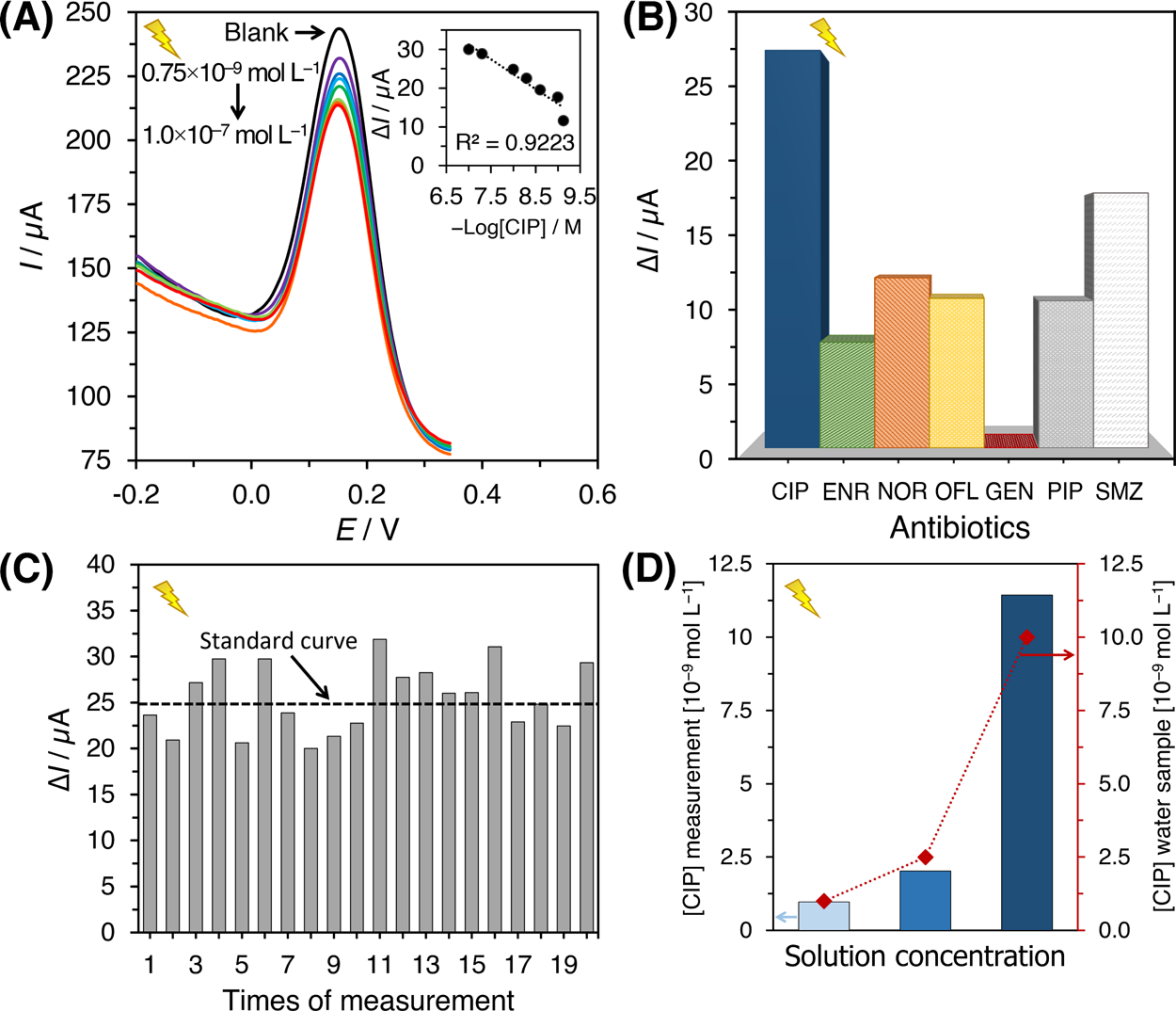
DPV curves corresponding to CIP concentrations (A); Δ*I* vs –Log[CIP] (*n* = 10) (A, Inset); selectivity of the sensor against CIP and other antibiotics (B); sensing responses of a single sensor to 1.0 × 10^–8^ mol L^–1^ CIP (C); and measurements at different concentrations of CIP in spiked actual water samples with the 0.25:0.25:1.00 CIMMO/rGO/GCE sensor (D).

The 0.25:0.25:1.00 CIMMO/rGO/GCE was incubated with the CIP solution at pH 6.5 for 1 min and then positioned in the electrochemical cell for the CIP measurement. The modified GCE was operated in DPV mode and exhibited sensitivity to CIP within a concentration range of 0.75 × 10^–9^–1.0 × 10^–7^ mol L^–1^ (Figure 5A). The current signal (Δ*I*) peaked at 0.16 V. We conducted a study to detect CIP concentrations across a wide range, which examined frequent measurements made at low concentrations and a rapid increase at high concentrations. The current signal demonstrated nonlinear behavior in response to the CIP concentrations and linear behavior with respect to the logarithmic CIP concentration (log[CIP]). The equation was Δ*I* (μA) = 7.68 log[CIP] (mol L^–1^) + 85.01, with an *R*^2^ of 0.9223 (*n* = 10), where Δ*I* denotes the difference in peak current in the absence and presence of CIP. The LOD was determined to be 4.74 × 10^–10^ mol L^–1^, based on the relationship LOD = 3.3 SD/S,^49^ where SD denotes the standard deviation of the intercept and S denotes the slope of the calibration plot (Figure 5A, inset). The logarithmic correlation between the electrochemical current and [CIP] suggested an indirect interaction between CIP and the composite. The peak intensity decreases as the CIP either competes with the mediator in withdrawing electrons from the working electrode, adsorbs onto and passivates the electrode, or interacts with the active mediator. We compared CIP sensing results from the composite sensor to the results of other reports (Section 2.5 and Table 1). Our results provided a low detection limit (0.00047 μmol L^–1^) and a wide linearity range (0.00075–0.10 μmol L^–1^). The 0.25:0.25:1.00 CIMMO/rGO/GCE sensor was selected for the cross-sensitivity (selectivity) test, as demonstrated in Figure 5B. The sensor was introduced to two groups of antibiotics: molecules structurally similar to CIP and molecules not structurally related at 1.0 × 10^–7^ mol L^–1^. The former group included enrofloxacin (ENR), norfloxacin (NOR), and ofloxacin (OFL), and the latter included gentamicin (GEN), piperacillin sodium salt (PIP), and sulfamethoxazole (SMZ). The sensor exhibited an Δ*I* value of 27.8 μA for CIP. The Δ*I*_CIP_ values were 3.76, 2.34, 2.66, 2.70, and 1.64 times higher than the responses for ENR, NOR, OFL, PIP, and SMZ, respectively, with no significant response against GEN.

**Table 1:** Comparative Analysis of the 0.25:0.25:1.00 CIMMO/rGO Sensor and Other Reported CIP Sensors

sensitive element	mode of detection	supporting electrolyte	pH of the supporting electrolyte	linearity range (μmol L^–1^)	limit of detection (μmol L^–1^)	ref
Au/C_3_N_4_/GN/GCE	SWV	PBS	7.0	0.6–120.0	0.42	(54)
Ch-AuMIP/GCE	DPV	PBS and K_3_Fe(CN)_6_	7.4	1.0–100.0	0.21	(55)
TiO_2_/PVA/GCE	DPV	PBS	7.0	10.0–120.0	0.04	(49)
Co-MOFs/PLA/GCE	DPV	PBS	7.0	0.5–150.0	0.017	(56)
MgFe_2_O_4_-MWCNTs/GCE	CV	PBS	3.0	0.10–1000.0	0.01	(57)
NH_2_–UiO-66/rGO/GCE	ASV	PBS	4.0	0.02–1.0	0.00667	(58)
Fe@g-C_3_N_4_/PGE	DPV	PBS	7.0	0.001–1.0	0.0054	(59)
CIMMO/rGO/GCE	DPV	PBS and [Fe(CN)_6_]^3–/4–^	6.5	0.00075–0.10	0.00047	this study

#### 2.3.3 Assessment of the Reusability, Reproducibility, and Stability of the Sensor

The reusability of the sensor was determined using the same modified electrode for successive DPV detection. The single modified electrode was introduced to 1.0 × 10^–8^ mol L^–1^ CIP in 0.05 mol L^–1^ PBS and analyzed in the [Fe(CN)_6_]^3–/4–^ medium (Figure 5C). The electrode was rinsed in DI water for 1.0 min and reintroduced into the CIP solution. This process was repeated 20 times, and the SD and relative standard deviation (RSD) for the measurements were determined to be 3.7 and 14.5%, respectively. The surface morphology of the CIMMO/rGO composite was analyzed by field emission-scanning electron microscopy (FE**-**SEM). The SEM showed no significant change in CIMM/rGO surface morphology from before to after one round of CV analysis (Figure S4A,B). The variations in sensing readouts could be attributed to the composite film damage caused by the surface tension of the solutions. We believe that the issue could be alleviated by coating another porous membrane, such as Nafion, on top of the composite film to promote film adhesion to the GCE and improve the physical strength. The reproducibility and stability of 0.25:0.25:1.00 CIMMO/rGO were evaluated in a mediator (Figure S5). The five electrodes, prepared using the same method, demonstrated good reproducibility with an RSD of their response currents of 1.10%. The stability of a single electrode was also assessed, yielding an RSD of 0.60%. Consequently, 0.25:0.25:1.00 CIMMO/rGO demonstrates commendable reproducibility and stability.

#### 2.3.4 CIP Detection in Surface Water Samples

The process used for water sample preparation is explained in the Supporting Information. Basic properties, including pH, temperature during sample collection, dissolved oxygen, conductivity, and salinity, were tested and are presented in Table S1. CIP was added to the surface water, creating solutions of 1.0 × 10^–9^, 2.5 × 10^–9^, and 10.0 × 10^–9^ mol L^–1^ CIP for the measurements. The readout values from the composite sensor were 1.0 × 10^–9^, 2.0 × 10^–9^, and 11.4 × 10^–9^ mol L^–1^, respectively, with *P* > 0.05 in the *t*-test analysis (Figure 5D). The deviations of the measurement were determined using the equation [(Sensor readout) – (Actual concentration)]/[Actual concentration] × 100 and were from 14 to 19% (Table S2).

### 2.4 Proposed Reaction Mechanisms

**Figure 6:**
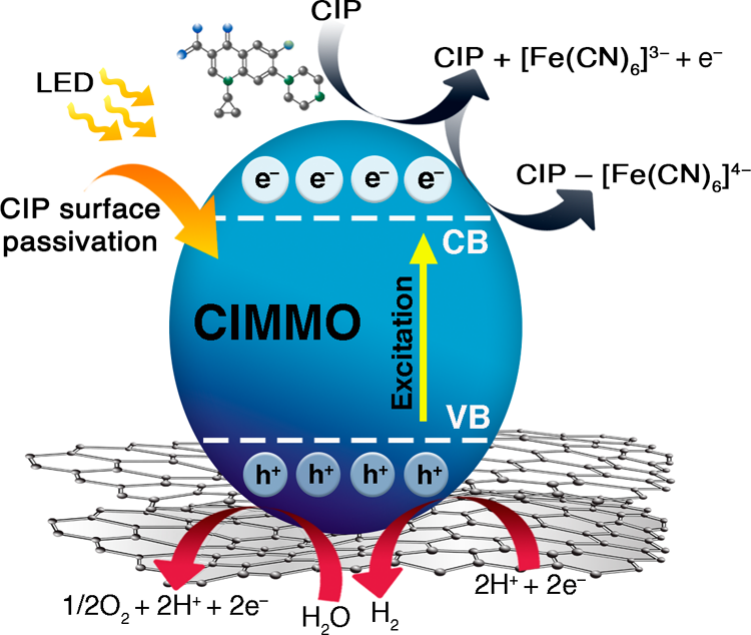
Schematic diagram showing proposed mechanisms for the indirect interactions of CIMMO/rGO with CIP.

The reaction mechanisms for the composite sensor rely on the types of metals, surface morphology, and interaction/reaction with the targeted analyte (Figure 6). As the CIMMO/rGO composite is illuminated, photoinduced electrons and holes (h^+^) are generated and repositioned at the conduction band (CB) and valence band (VB), respectively (eq 4). The electron–hole separation occurs with partial assistance from the applied potential at the electrode. CIP oxidation means that electrons withdraw from the working electrode, which should result in a higher anodic current. However, the response from the DPV technique decreased as the CIP concentration increased, suggesting diminishing electron transfer at the working electrode. The [Fe(CN)_6_]^3–^ was electrochemically reduced to [Fe(CN)_6_]^4–^, establishing the redox couple [Fe(CN)_6_]^3–^/[Fe(CN)_6_]^4–^. The reduction in current could be attributed to the interactions of CIP and [Fe(CN)_6_]^3–/4–^ (eq 5), which decelerated the charge transfer or CIP adsorption that partly passivated the CIMMO/rGO electrode (eq 6).^50^ It is worth noting that both phenomena involve an equal number of electron and proton transfers (Section 2.2.3 and Figure 4D), indicating the indirect interactions between the CIP and the working electrode.
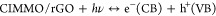
4
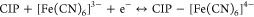
5
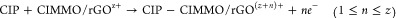
6
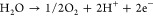
7
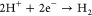
8
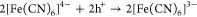
9
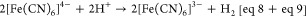
10

The reduction reaction completing the redox reaction occurs at the composite CB. It involves water (H_2_O) dissociation into oxygen, electrons, and protons (eq 7), giving two electrons (*n* = 2) for the electrochemical reaction.^51^ The protons can couple to other electrons to produce H_2_ (eq 8). Photoinduced holes (h^+^) interact with [Fe(CN)_6_]^3–^ mediator, yielding [Fe(CN)_6_]^4–^ (eq 9). The total reaction (eq 8 + eq 9) is presented in eq 10.^52^ The combination of mixed metal oxides in the composite structures resulted in a synergistic effect, in which the iron oxides contributed mainly as the photoelectrocatalyst.^53^ As the composite was illuminated, the electron energy was enhanced from the VB to the CB level. The CB electrons crossed the copper–iron interfaces, reaching the copper metal oxide matrix with an effective electron transfer ability. The CIP accepted photoinduced electrons from the copper oxide sites (CB), while the photoinduced h^+^ were withdrawn from the VB site of iron and copper oxides. The synergy phenomenon enhanced the number of photoinduced electrons by reducing the rate of electron–hole recombination, promoting the photoactivity of the composite material.

### 2.5 Comparative Analysis between the CIMMO/rGO Electrochemical Sensor and Other Reported Sensors

We compared the 0.25:0.25:1.00 CIMMO/rGO/GCE sensor with other reported sensors (Table 1). Yuan et al.^54^ synthesized Au nanoparticles/carbon nitride/graphene (Au/C_3_N_4_/GN/GCE) and cast it onto GCE. The electrode, operated in square wave voltammetry (SWV) mode, exhibited a linear detection window from 0.60 to 120.0 μmol L^–1^ CIP and an LOD of 0.42 μmol L^–1^. Surya et al.^55^ combined gold nanoparticles and chitosan to create MIP on a GCE (Ch-AuMIP/GCE). The MIP electrode was operated in DPV mode and at concentrations of 1.0–100.0 μmol L^–1^ CIP. The LOD value was reported as 0.21 μmol L^–1^. Zhao et al.^49^ coated TiO_2_ and poly(vinyl alcohol) onto a GCE (TiO_2_/PVA/GCE) using a dip-coating technique. They obtained a broad and good detection response from CIP with a linear range of 10.0–120.0 μmol L^–1^ and LOD of 0.04 μmol L^–1^. Yahyapour et al.^56^ synthesized cobalt (Co)-metal-organic frameworks (MOFs) and polylactic acid nanofiber on GCE (Co-MOFs/PLA/GCE). The Co-MOF/PLA/GCE had a linear response range to CIP of 0.5–150.0 μmol L^–1^ and an LOD of 0.017 μmol L^–1^. Ensafi et al.^57^ prepared magnesium ferrite (MgFe_2_O_4_) nanoparticle-decorated multiwalled carbon nanotubes (MWCNTs) on GCE (MgFe_2_O_4_-MWCNTs/GCE) and studied the electrode using the CV technique. It showed good detection within a 0.10–1000.0 μmol L^–1^ CIP range with an LOD of 0.01 μmol L^–1^. Fang et al.^58^ prepared Zr(IV)-based MOF and rGO composite (NH_2_–UiO-66/rGO/GCE). The composite was fabricated with an electrochemical sensor that relied on anodic stripping voltammetry (ASV) mode. The sensor detected CIP within a linear range of 0.02–1.0 μmol L^–1^ and had an LOD of 0.00667 μmol L^–1^. Vedhavathi et al.^59^ modified a pencil graphite electrode (PGE) with an iron-decorated graphitic carbon nitride composite (Fe@g-C_3_N_4_/PGE). Operated in a DPV mode, the sensor showed a CIP linear detection range of 0.001–1.0 μmol L^–1^ and an LOD of 0.0054 μmol L^–1^. Compared to sensors described in previous reports, the composite sensor examined in this study demonstrated an enhanced efficacy for CIP detection, exhibiting an extensive linear range and a notably low LOD.

## 3 Conclusions

We synthesized CIMMO/rGO composites following a one-step hydrothermal technique and coated them as a thin film on a GCE electrode. The 0.25:0.25:1.00 CIMMO/rGO/GCE demonstrated outstanding performance as an electrochemical sensor for CIP detection, producing the equation of Δ*I* (μA) = 7.68 log[CIP] (mol L^–1^) + 85.01 (*R*^2^ of 0.9223) for 0.75 × 10^–9^–1.0 × 10^–7^ mol L^–1^ CIP. The LOD was 4.74 × 10^–10^ mol L^–1^. The composite sensor exhibited acceptable selectivity to CIP, providing responses to CIP that were 1.6 to 3.8 times higher than responses to other antibiotics. The sensor also showed good reusability and sufficient responses to CIP in actual water samples.

## 4 Methodology

### 4.1 Chemicals and Reagents

All chemicals were of analytical grade and used with no additional purification. The aqueous solutions were prepared with UPW from a Millipore Milli-Q system (18.2 MΩ·cm). Graphite flakes (10 mesh size) were purchased from Alfa Aesar (USA). Copper nitrate trihydrate (Cu(NO_3_)_2_·3H_2_O) (Qrec, New Zealand), potassium permanganate (KMnO_4_) (Ajax Finechem Pty, Ltd., Australia), iron(III) chloride (FeCl_3_) (Carlo Erba, Italy), and sodium nitrate (NaNO_3_) (Fluka Chemika, Switzerland) were used as received. Hydrazine sulfate (H_2_N–NH_2_·H_2_SO_4_) (AppliChem GmbH, Germany) and sulfuric acid (H_2_SO_4_) (RCI Labscan, Ltd., Thailand) were reagent grade and used with no further treatment. CIP, ENR, NOR, OFL, SMZ, and PIP were HPLC grade and purchased from Sigma-Aldrich (USA). GEN was purchased from Himedia Laboratories Pvt. Ltd. (India). Potassium hexacyanoferrate (III) (K_3_Fe(CN)_6_), potassium hexacyanoferrate (II) trihydrate (K_4_Fe(CN)_6_·3H_2_O), and potassium chloride (KCl) were obtained from Sigma-Aldrich (USA). Ethanol (C_2_H_5_OH), 30% hydrogen peroxide (H_2_O_2_), hydrochloric acid (HCl), sodium borohydride (NaBH_4_), and sodium hydroxide (NaOH) were purchased from Merck, Germany. Dipotassium phosphate (K_2_HPO_4_) and potassium phosphate (KH_2_PO_4_) (Merck, Germany) were used in the preparation of 0.05 mol L^–1^ PBS.

### 4.2 GO Synthesis

GO was chemically synthesized following a chemical exfoliation method.^60^ We mixed 2.0 g of graphite flakes with 1.0 g of NaNO_3_ and 50.0 mL of H_2_SO_4_ and stirred the mixture for 2 h at 0 °C. The KMnO_4_ oxidizer (7.3 g) was gradually poured into the mixture during the stirring. The mixture was moved to ambient conditions and stirred for 2.5 h before a solution of 55.0 mL of UPW and 7.0 mL of H_2_O_2_ was added to terminate the oxidation reaction. The powder was rinsed with 3% HCl and UPW to remove excess manganese residuals. GO was heated in a vacuum oven at 60 °C for 24 h and stored in a desiccator until used.

### 4.3 Synthesis of the CIMMO/rGO Composite

**Figure 7:**
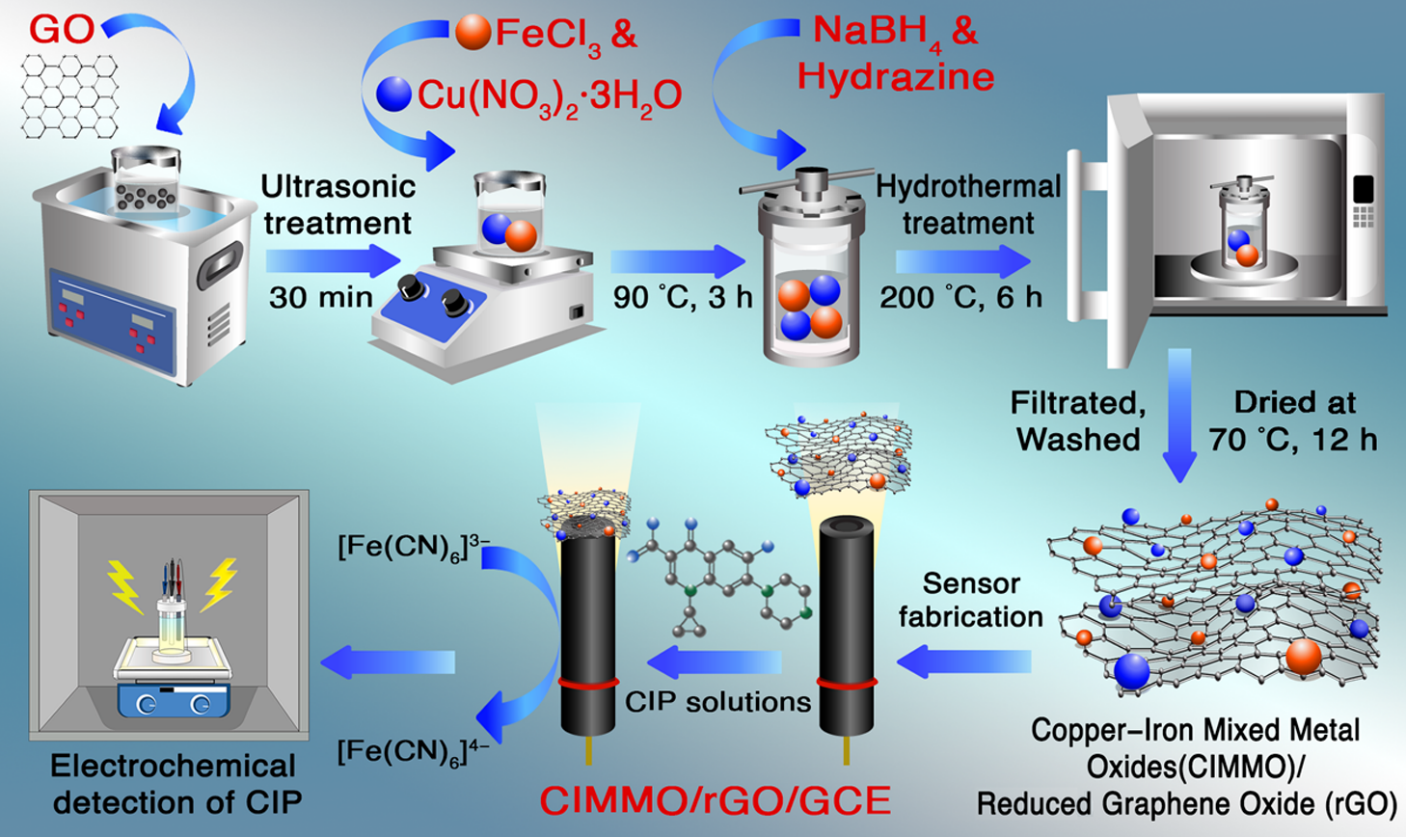
Diagram showing the CIMMO/rGO/GCE fabrication process and CIP detection.

The CIMMO/rGO composites were synthesized using a hydrothermal technique modified from the method presented by Yang et al.^61^ GO suspension was prepared in distilled water with a known amount of Cu(NO_3_)_2_·3H_2_O and FeCl_3_, which was added with stirring at 90 °C for 3 h (Figure 7). The mixture was cooled to ambient temperature and mixed with hydrazine before being transferred to a 100 mL Teflon-lined autoclave. The autoclave was held at 200 °C for 6 h. The composite powder was rinsed several times using UPW and dried at 70 °C to obtain fine particles. GO was introduced to hydrazine with heat during composite production and was reduced to rGO in the CIMMO/rGO composites. The composite was prepared at various Cu:Fe:GO ratios, among which the ratio of 0.25:0.00:1.00 indicates a composite that was synthesized using 0.25 w/w copper mixed metal oxide precursor/GO, 0.00 w/w iron mixed metal oxide precursor/GO, and 1.00 w/w GO/GO. Composites with other intermediate ratios, including 0.00:0.25:1.00, 0.25:0.25:1.00, 1.00:1.00:1.00, 1.50:1.50:1.00, and 2.50:2.50:1.00, were also synthesized and characterized.

### 4.4 Fabrication of CIMMO/rGO/GCE

The composite powder was dispersed in UPW (1.0 mg mL^–1^) via ultrasonication at 59 W, creating the suspension. The GCE was polished sequentially with 1.0, 0.3, and 0.05 μm alumina slurries, followed by treatment with 1:1 (v/v) HNO_3_/UPW, ethanol, and UPW. The cleansed surface was dried in a stream of nitrogen. A 10-μL drop of CIMMO/rGO suspension was cast on the GCE and warmed at 60 °C for 15 min to dry. The composite film was uniform at 1.42 μg of mm^–2^.

### 4.5 Electrochemical Measurements

A 3-electrode electrochemical quartz cell employed a GCE (working electrode) with an Ag/AgCl reference electrode and platinum mesh (counter electrode). Electrochemical signals were monitored through a portable PalmSens4 analyzer under intense irradiation of four light-emitting diodes (LEDs) (3W, 320 lm/bulb). The sensors were operated in CV mode with a −0.2 to +0.6 V potential window and 0.05 V s^–1^ scan rate for 10 cycles. The sensor was used in DPV mode, with the application of a wide potential window of −0.2 to +0.6 V, step potential (*E*_step_) of 0.005 V, pulse potential (*E*_pulse_) of 0.06 V, pulse time (*t*_pulse_) of 0.02 s, and scan rate of 0.05 V s^–1^. We used 5.0 × 10^–3^ mol L^–1^ [Fe(CN)_6_]^3–/4–^ redox mediator in 0.1 mol L^–1^ KCl at pH 6.5 as a medium for electrochemical measurements. EIS measurements were performed at 0.1 to 100,000.0 Hz with a DC potential of +0.15 V and a set amplitude (*E*_ac_) of +0.006 V. The EIS analysis was carried out using a modeled equivalent circuit.

### 4.6 Material Characterizations

μXRF (XGT-9000, Horiba, Ltd., Japan) was utilized to determine the mass composition of the samples. To analyze crystal structures, XRD (D2 Phaser, Bruker, Germany, CuKα radiation) was performed with a scanning range from 5° to 90° (2θ) at a step size of 0.02° and a scanning rate of 2° min^–1^. FT-IR analyses were recorded on a Nicolet iS50 spectrometer in the 400–4000 cm^–1^ range. BET analysis (Quantachrome ASIQwin, Automated gas sorption analyzer instrument, version 5.23) was used for specific surface area and pore volume analysis. Physical morphologies of the samples were observed by using TEM (FEI, Tecnai G2 20) operated at 200 kV and FE-SEM (JEOL Ltd., JSM-7800F Prime instrument, Tokyo, Japan). Surface compositions and chemical binding energies were studied by using XPS (Kratos AXIS Ultra DLD, Kratos, UK), utilizing a monochromatized Al Kα radiation source.

## Supporting Information Available

Additional information as noted in the text; preparation of actual water samples; basic properties of surface water; actual water sample testing and statistical analysis; size distribution histograms; XPS survey spectra; magnetic characteristic of the various ratio of CIMMO/rGO composite and the effect of medium pH on sensing responses; FE-SEM images; and the reproducibility and the stability of sensor (PDF)

## Abbreviations

ABR: antibiotic resistance
ASV: anodic stripping voltammetry
BET: Brunauer–Emmett–Teller
BJH: Barrett–Joyner–Halenda
CB: conduction band
CIMMO: copper–iron mixed metal oxides
CIP: ciprofloxacin
Cu: copper
CV: cyclic voltammetry
DPV: differential pulse voltammetry
EBSD: electron backscatter diffraction mode
EIS: electrochemical impedance spectroscopy
ENR: enrofloxacin
Fe: iron
GC: gas chromatography
GCE: glassy carbon electrode
GEN: gentamicin
GO: graphene oxide
HPLC–MS/MS: high-performance liquid chromatography coupled with mass spectrometry
IUPAC: International Union of Pure and Applied Chemistry
LEDs: light-emitting diodes
LOD: limit of detection
MIPs: molecularly imprinted polymers
MMWCNTs: magnetic multiwalled carbon nanotubes
MWCNTs: multiwalled carbon nanotubes
NOR: norfloxacin
OFL: ofloxacin
PBS: phosphate buffer solution
PGE: pencil graphite electrode
PIP: piperacillin sodium salt
rGO: reduced graphene oxide
*R*_ct_: electron transfer resistance
RSD: relative standard deviation
SAED: selected area electron diffraction
SD: standard deviation
SMZ: sulfamethoxazole
SWV: square wave voltammetry
UPW: ultrapure water
VB: valence band
