# Correction to “Ciprofloxacin Electrochemical Sensor Using Copper–Iron Mixed Metal Oxides Nanoparticles/Reduced Graphene Oxide Composite”

**DOI:** 10.1021/acsomega.5c03624

**Published:** 2025-05-06

**Authors:** Jedsada Chuiprasert, Sira Srinives, Narin Boontanon, Chongrak Polprasert, Nudjarin Ramungul, Apisit Karawek, Suwanna Kitpati Boontanon

In the original article, the funding statement in the Acknowledgments section is not comprehensive and should be revised as shown below to include the complete funding information.

