# Supplementary material for: Ciprofloxacin Electrochemical Sensor Using Copper–Iron Mixed Metal Oxides Nanoparticles/Reduced Graphene Oxide Composite
Source: ACS Omega. 2024 May 21;9(22):23172–83. doi: 10.1021/acsomega.3c06705 (PMC11166261; doi:10.1021/acsomega.3c06705)
Supplement: Supplementary file 1 — ao3c06705_si_001.pdf [file ao3c06705_si_001.pdf]

Supporting Information

Ciprofloxacin Electrochemical Sensor Using Copper−Iron Mixed Metal Oxides Nanoparticles/Reduced Graphene Oxide Composite

Jedsada Chuiprasert,[†] Sira Srinives,[*,‡] Narin Boontanon,[§] Chongrak Polprasert,[∥] Nudjarin Ramungul,[⊥] Apisit Karawek,[‡] and Suwanna Kitpati Boontano[*,†,#]

[†]Graduate Program in Environmental and Water Resources Engineering, Department of Civil and Environmental Engineering, Faculty of Engineering, Mahidol University, Salaya, Phuttamonthon, Nakhon Pathom 73170, Thailand

[‡]Nanocomposite Engineering Laboratory (NanoCEN), Department of Chemical Engineering, Faculty of Engineering, Mahidol University, Salaya, Phuttamonthon, Nakhon Pathom 73170, Thailand

[§]Faculty of Environment and Resource Studies, Mahidol University, Salaya, Phuttamonthon, Nakhon Pathom 73170, Thailand

[∥]Department of Civil Engineering, Faculty of Engineering, Thammasat University, Khlong Nueng, Khlong Luang, Pathum Thani 12120, Thailand

[⊥]National Metal and Materials Technology Center, National Science and Technology Development Agency, Khlong Nueng, Khlong Luang, Pathum Thani 12120, Thailand

[#]Graduate School of Global Environmental Studies, Kyoto University, Yoshida-Honmachi, Sakyo-ku, Kyoto 606-8501, Japan

***Corresponding Author Email:**

suwanna.boo@mahidol.ac.th, (Suwanna Kitpati Boontanon) and
sira.sri@mahidol.edu, (Sira Srinives)

1. Preparation of actual water samples.

We collected surface water samples at Mahidol University, Nakhon Pathom, Thailand, on April 8th, 2022, and filtrated the water using a 1.0 μm membrane filtration apparatus. The water was mixed with the 0.05 mol L^{-1} phosphate buffer solution (PBS) at a 1:5 (v/v) ratio and ciprofloxacin (CIP) solution to attain a designed concentration.

Table S1 Basic properties monitored from the surface water

Parameters	Water quality
pH	8.11 ± 0.01
Temperature (°C)	25.5
Dissolved oxygen (mg L^{-1})	0.6
Conductivity (mS cm^{-1})	0.75 ± 0.03
Salinity (g L^{-1})	0

Table S2 Results of actual water sample testing and statistical analysis using the 0.25:0.25:1.00 CIMMO/rGO/GCE sensor

Sample	Added concentration (mol L^{-1})	Measured concentration (mol L^{-1})	Deviation (%)
Surface water	1.0×10^{-9}	1.0×10^{-9}	−2.88
	2.5×10^{-9}	2.0×10^{-9}	−19.08
	10.0×10^{-9}	11.4×10^{-9}	14.38

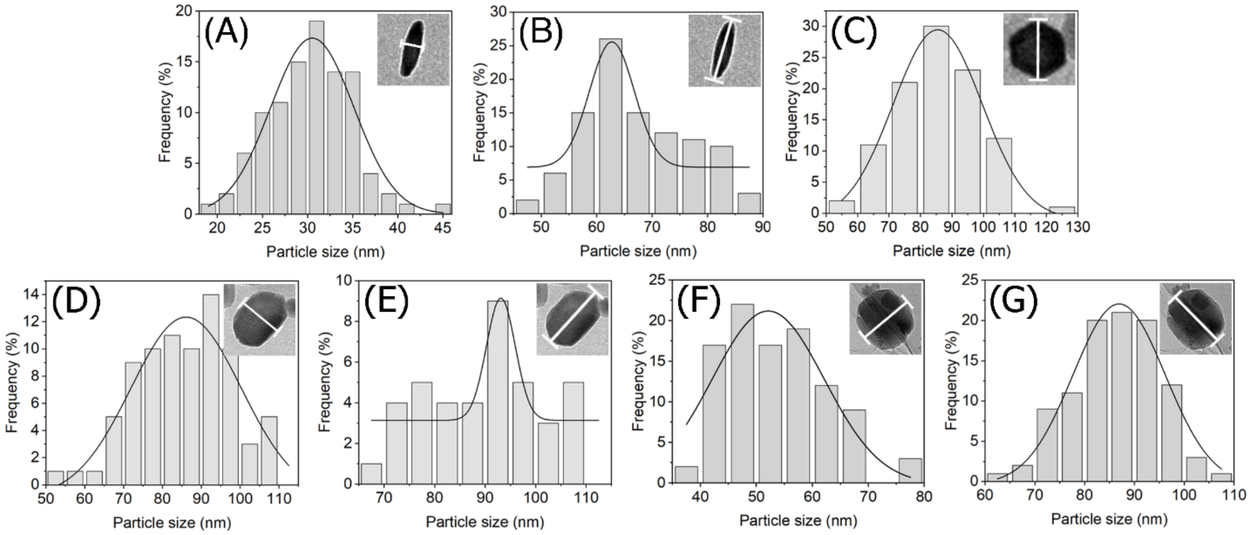

Figure S1 Size distribution histograms with Gaussian-fitting curves extracted from TEM images, showing CuO and Cu_2O widths (A) and lengths (B); α−Fe_2O_3 octadecahedral (C) and rhombohedral widths (D) and lengths (E); and $CuFe_2O_4$ irregular spherical widths (F) and lengths (G).

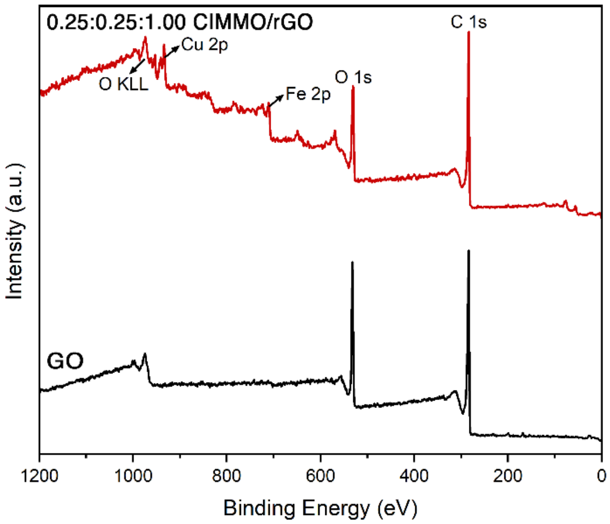

Figure S2 XPS survey spectra of the GO and the 0.25:0.25:1.00 CIMMO/rGO.

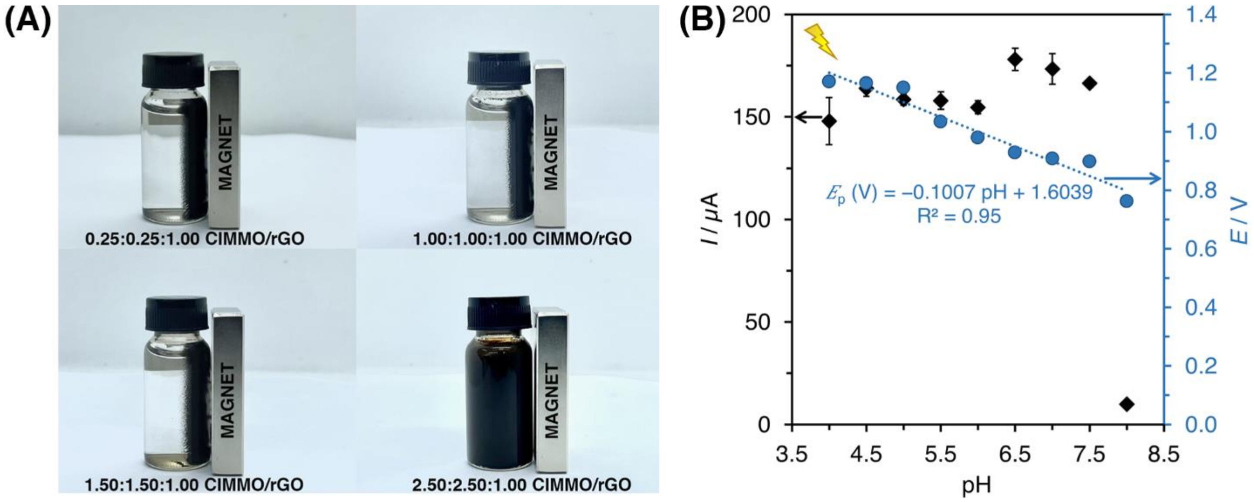

Figure S3 Demonstration of magnetic properties of CIMMO/rGO composites (A) and the effect of medium pH on sensing responses against 1.0 × 10^{-6} mol L^{-1} CIP (B).

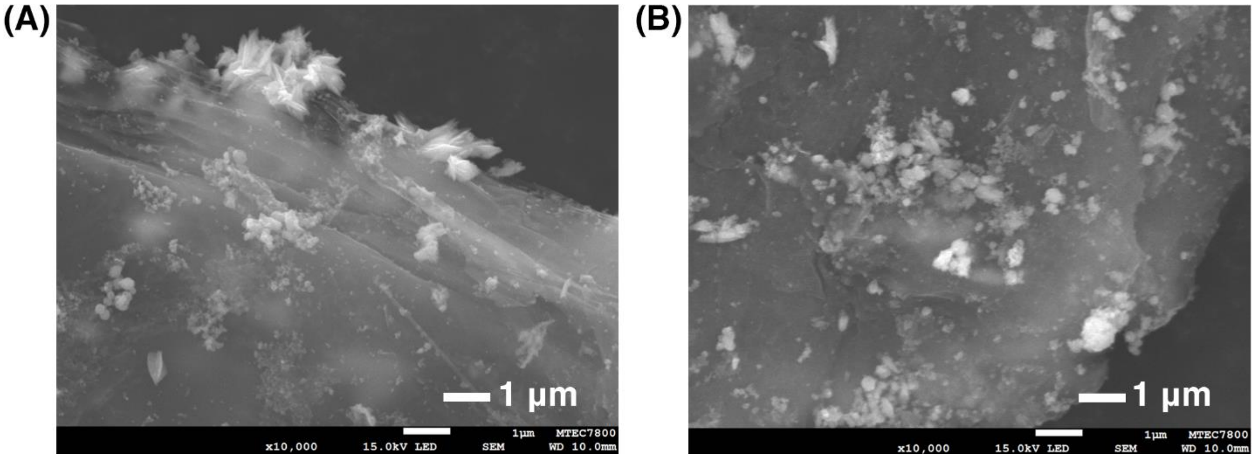

Figure S4 FE-SEM images of 0.25:0.25:1.00 CIMMO/rGO composite as before (A) and after (B) one round of CV analysis.

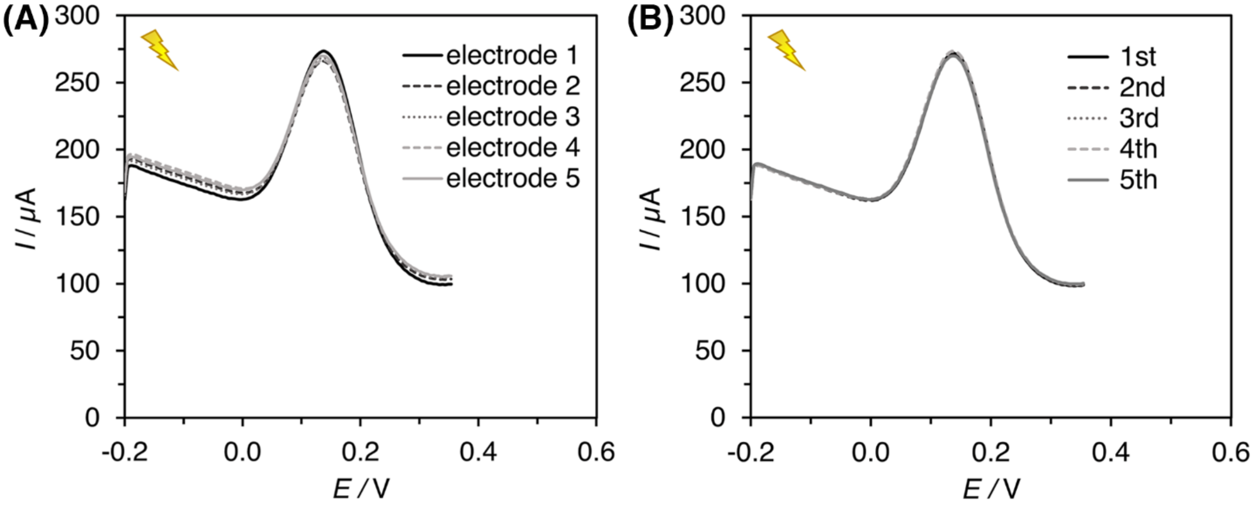

Figure S5 The reproducibility of five 0.25:0.25:1.00 CIMMO/rGO electrodes prepared using the same method (A) and the stability of responses from a single electrode tested five times (B) in 5.0×10^{-3} mol L^{-1} $[Fe(CN)_6]^{3-/4-}$ redox mediator in 0.1 mol L^{-1} KCl.
